# Methods to Promote Germination of Dormant *Setaria viridis* Seeds

**DOI:** 10.1371/journal.pone.0095109

**Published:** 2014-04-18

**Authors:** Jose Sebastian, Mandy Ka Wong, Evan Tang, José R. Dinneny

**Affiliations:** Carnegie Institution for Science, Department of Plant Biology, Stanford, California, United States of America; National Institute of Plant Genome Research, India

## Abstract

*Setaria viridis* has recently emerged as a promising genetic model system to study diverse aspects of monocot biology. While the post-germination life cycle of *S*. *viridis* is approximately 8 weeks long, the prolonged dormancy of freshly harvested seeds can more than double the total time required between successive generations. Here we describe methods that promote seed germination in *S*. *viridis*. Our results demonstrate that treating *S*. *viridis* seeds with liquid smoke or a GA_3_ and KNO_3_ solution improves germination rates to 90% or higher even in seeds that are 6 days post-harvest with similar results obtained whether seeds are planted in soil or on gel-based media. Importantly, we show that these treatments have no significant effect on the growth of the adult plant. We have tested these treatments on diverse *S*. *viridis* accessions and show variation in their response. The methods described here will help advance research using this model grass species by increasing the pace at which successive generations of plants can be analyzed.

## Introduction

Monocots contribute the largest share of the world's food supply and biofuel feed stock (http://faostat.fao.org/site/339/default.aspx). Understanding biological processes and pathways that are specific to monocots is therefore of great importance from an applied science perspective and also for understanding the diversity of biological processes that occurs in flowering plants. *Setaria viridis* (*S. viridis*) is a grass species in the Panicoideae clade of the Poaceae family commonly known as green foxtail. *S. viridis* is an invasive weed that can be found worldwide [1]. An array of *S. viridis* traits make this plant species highly useful as a model for understanding bioenergy grasses including its small plant size (20–50 cm), relatively small genome (∼515 Mb), fast lifecycle (6–8 weeks), prolific seed production, ease of growing under controlled conditions and C_4_-type photosynthesis pathway. *S. viridis* has recently been used to study a range of biological processes from flowering time, shoot architecture and the evolution of photosynthesis [2]–[5]. The *S. viridis* accession A10, whose draft whole-genome sequence was recently completed, is currently used as the reference accession [6], [7]. In order to improve the utility of *S. viridis* as an experimental model for molecular genetic studies, stable transformation protocols are being developed in addition to the development of methods to perform directed crosses [8]. Despite the rapid development of *S. viridis*, extensive seed dormancy makes it difficult to take advantage of the short lifecycle. Several studies have reported the extent of dormancy in *S. viridis* seeds and it is widely considered as an adaptation enhancing its growth and survival as a weed species [9]–[13].

Seed dormancy prevents germination of the seedling and may be the result of several different physiological and developmental mechanisms (Reviewed by [14]), [15], [16]. In nature, several factors can often act together to prevent germination until a favorable condition arises. Freshly collected *S. viridis* seeds often show close to 0% germination rates when planted in soil or in tissue culture [9]. It was reported that the mean germination rate gradually increases from 0 to 42% over 55 days after seed harvest and up to 56% after 115 days [9]. Of the various treatments previously employed to break seed dormancy in *S. viridis*, none was found promising except moist storage of seeds at 6°C for 3 to 6 weeks. The considerable length of time required for breaking dormancy delays further experimentation and the development of genetic resources such as Recombinant Inbred Lines (RILs) for Quantitative trait loci (QTL) analysis, which often require 6–8 generations of selfing.

In this study we searched for an efficient method to break dormancy in *S. viridis* seeds and tested a range of chemical treatments to establish an optimized protocol. Our studies highlight liquid smoke and gibberellic acid (GA_3_) as germination stimulants, which can promote 90 percent or higher seed germination rates in *S. viridis* seeds that are as young as 6 days post-harvest. This improved protocol substantially increases the efficacy of *S. viridis* as a model molecular genetic system.

## Materials and Methods

### Plant materials and experimental conditions

*S*. *viridis* reference accession A10 seeds were used in this study unless otherwise described. Mature seeds were collected from plants grown in a growth chamber (12 hours light at 29°C and 12 hours dark at 24°C with constant relative humidity at 54–55%) or green house (12 hours light at 31°C and 12 hours day at 23°C, humidity not controlled). Other *S. viridis* accessions were obtained from the USDA (North Central Regional Plant Introduction Station, Iowa State University) or from the lab of Tom Brutnell at the Donald Danforth Plant Science Center (St. Louis, MO). Accessions were subsequently propagated and used for dormancy analyses. After harvesting, seeds were dried in paper bags at room temperature until their use. Dead or aborted seeds were manually culled and not used in experiments. For various treatments, seeds were treated with different concentrations of chemicals in an aqueous solution in an eppendorf tube, vortexed briefly, and incubated as described in the main text. Treated seeds were subsequently sown onto soil (Pro-Mix) imbibed in water and kept in a growth chamber (12 hours light at 31°C and 12 hours dark at 23°C with constant relative humidity at 54–55%). For analysis of germination rates in tissue culture, treated seeds were plated on MS gelrite media (0.5X MS, 0.5% sucrose, pH 5.7) with 0.7% gelrite (Gelzan, Sigma, catalog# G1910) in 90 mm^2^ square petri dishes.

### Preparation of seed treatment solutions

Gibberellic acid (Sigma, Catalog# G7645) was initially dissolved in 95% ethanol to make a primary stock solution of 200 mM and stored at −20°C. The working concentrations of GA_3_ were made fresh each time in distilled water and 30 mM KNO_3_ was added to it from a stock solution of 500 mM. For liquid smoke (concentrated solutions were purchased from a local supermarket), diluted solutions were made each time prior to seed treatment from the concentrated commercial liquid smoke solution. Karrikinolide (3-methyl-2H-furo(2,3-c)pyran-2-one (karrikin-1), obtained from the lab of Winslow Briggs, Department of Plant Biology, Carnegie Institution for Science), was initially dissolved in 95% ethanol to make a primary stock solution of 2 mM and stored at −20°C. The working concentrations of karrikinolide were made fresh before use in distilled water. Fluridone (Fluka, Catalog# 45511) was dissolved in water to make a primary stock solution of 100 mM and stored at −20°C and working concentrations were made fresh before use. After each treatment, seeds were either directly sown onto soil or sterilized (as mentioned below) and plated onto tissue culture plates.

### Germination assays

Rates of seed germination were determined 8 days after sowing (DAS) seeds to soil. Appearance of the coleptile was used to mark a germinated seedling. To quantify germination rates in tissue culture, germination was quantified 3 days after plating. Here, protrusion of the radicle was considered as the criterion for germination.

### Sterilization and growth of seeds on MS gelrite plates

Seeds were treated with 20% commercial bleach solution containing 0.1% of Tween-20 for 20 minutes with occasional vortexing. Seeds were subsequently washed 3 to 4 times with sterile distilled water. After sterilization, seeds were placed on the surface of MS gelrite media and plates sealed with micropore tape. Plates were incubated in a plant growth chamber (Percival) at 12 hours light at 29°C and 12 hours dark at 24°C.

### Statistical analyses

6–8 seeds were planted per pot and three to eight pots were used per treatment/condition. Germination rates were quantified per pot and averaged across all the pots and error calculated. All experiments were repeated three times with similar results, except the survey of *S. viridis* accessions, which was performed two times. The mean and standard error for measurements were calculated for leaf number (at the time of flowering), flowering time (day when panicle became visible), tiller number, panicle size and the number of crown roots. Statistical significance of results was calculated using Student's T-test with a *P*-value threshold of less than 0.05.

## Results

### Identification of treatments that break seed dormancy in *S. viridis*

**Figure 1:**
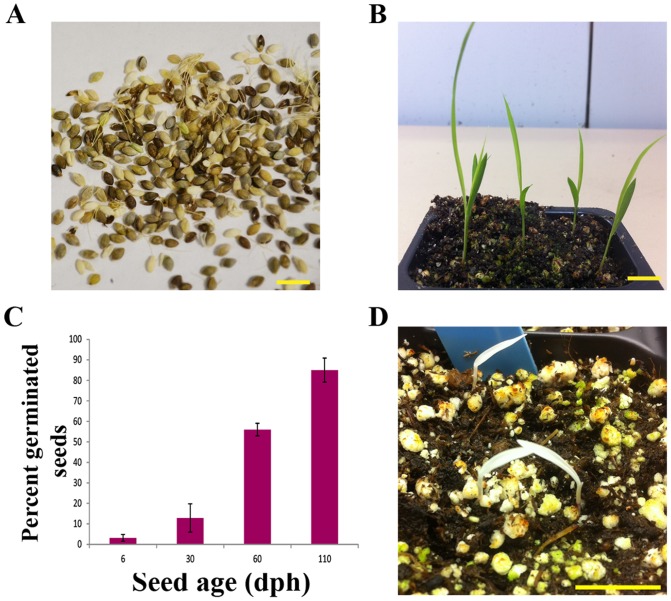
*S. viridis* seeds show extensive dormancy. (**A**) *S. viridis* seeds. (**B**) *S. viridis* seedlings at 8 DAS. (**C**) Extent of seed dormancy in *S. viridis* seeds (n = ≥ 25 seeds per replicate). (**D**) Albino seedlings from *S. viridis* seeds treated with fluridone. Scale bars are A, 5 mm; B & C, 15 mm. Error bars represent standard error.

To investigate the extent of seed dormancy in *S. viridis*, seeds were harvested at different time points (ranging from 6 days to 110 days post-harvest) and sown in moist soil (Figure 1A & B). Dormancy in *S. viridis* seeds is dependent on the length of time post-harvest (days post-harvest, dph) and germination rates improved above 50% only after 60 dph (Figure 1C). To identify chemicals and/or conditions that increased germination rates, one month old *S. viridis* seeds were exposed to varying concentrations of different chemicals known to aid in breaking seed dormancy in other plant species [17]–[23]. We also tested temperature shock treatments or removing the lemma and palea in order to eliminate any mechanical hindrance. A detailed list of chemicals and conditions employed are given in the Table S1.

Among these conditions tested, treatment of seeds with fluridone, GA_3_, liquid smoke and karrikinolide had the greatest effect on dormancy in freshly collected *S. viridis* seeds. Fluridone inhibits an early step of the carotenoid biosynthesis pathway [24]. Treatment of seeds with fluridone improved germination rates dramatically; however, germinated seedlings appeared albino and failed to recover subsequently (Table S1, Figure 1D). GA_3_ + KNO_3_ and liquid smoke treatments had no adverse effects on plant growth and development, thus we selected these chemicals to test the effects of other treatment parameters. We analyzed the growth of plants from seedling stage to flowering to determine whether GA_3_ + KNO_3_ and liquid smoke treatments had any other effects. Multiple plant characteristics including leaf number at flowering, flowering time (time of panicle emergence), panicle size, plant height, tiller number, crown root number and leaf initiation rates were examined (Table 1, Figure S1). With all the methods tested, incubation of seeds at warmer temperatures (ranging from 29 to 31°C) appeared beneficial, while, low temperature (4°C) incubation for short durations was found to negatively affect seed germination. In addition, our results suggested that removing the lemma and palea had an inhibitory effect on seed germination in soil. Incubation of 30 dph seeds in moist soil at 4°C for 2 weeks improved germination to 55±3.5% germination compared to 15±8.7% in seeds stored at room temperature, similar to previous reports (Table S1). In our trials, prior cold stratification at 4°C or −80°C did not have an effect on germination rates. We have also tested the use of GA_3_, KNO_3_ and liquid smoke for breaking seed dormancy when sterilized seeds are grown on MS gelrite media. Treating one month old *S. viridis* seeds with 1.44 mM GA_3_ + 30 mM KNO_3_ (78±3.4%) or 1% liquid smoke (80±2.1%) at 29°C for 24 hours followed by sterilization and plating on to MS gelrite media increased the germination rates significantly compared to control seeds soaked in distilled water (8.6±3.8%) (Table S2).

**Table 1:** Phenotypic characterization of plants from seeds treated with GA_3_ and liquid smoke.

	5% LS	GA_3_ + KNO_3_	water
Leaf number at flowering	6.98±0.02*	7.06±0.03	6.97±0.07
Flowering time	22.4±0.29	22.6±0.25	22.5±0.28
Tiller number	2.57±0.22	2.6±0.21	2.69±0.30
Panicle size	2.78±0.07	2.73±0.08	2.77±0.08
Number of crown roots	2.60±0.14	2.82±0.14	2.75±0.20
Plant height	17.0±0.57	16.8±0.61	16.9±0.69

Tiller number, panicle size, crown root number and plant height were quantified at 24 DAS. Panicle size (from the tip of the panicle to its base) and plant height (from the tip of the panicle to the bottom of the stem) are measured in centimeters. Leaves on the main plant are only included for leaf number measurements. Flowering time is given as DAS. All visible tillers present on the main plant are considered for tiller counting. (n = 25 to 30 plants per replicate). LS, liquid smoke; GA_3_ + KNO_3_, 2.89 mM GA_3_ + 30 mM KNO_3_ in distilled water. * Represents the standard error.

### Effect of GA_3_ on dormancy in *S. viridis* seeds

**Figure 2:**
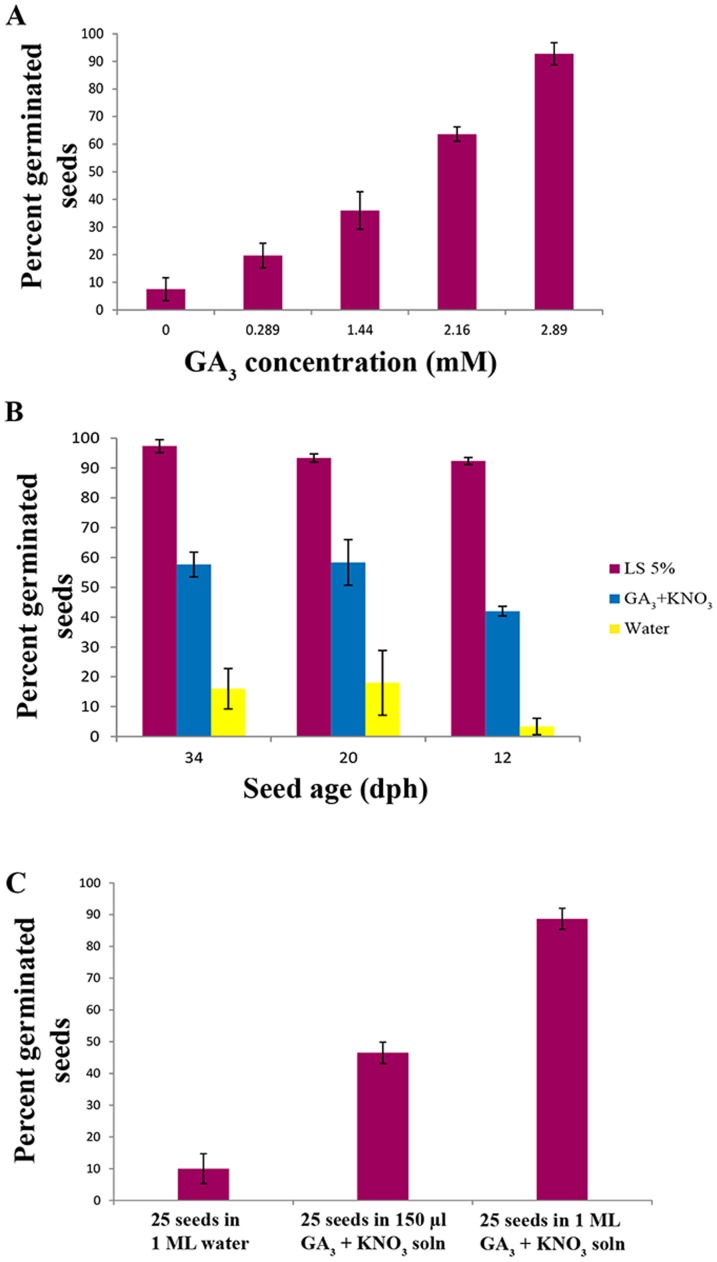
Effect of GA_3_ and liquid smoke on dormancy in *S. viridis* seeds. (**A**) Dose dependent effect of GA_3_ on promoting *S. viridis* seed germination (seed age 30 dph). (**B**) Effect of GA_3_ and liquid smoke on stimulating germination of *S. viridis* seeds of different ages (dph) (n = 50 seeds per replicate). (**C**) An apparent concentration effect of GA_3_ in promoting *S. viridis* seed germination (n = 25 to 30 seeds per replicate, seed age 30 dph). Error bars represent the standard error. LS, liquid smoke; dph, days post-harvesting; GA_3_ solution, 2.89 mM GA_3_ + 30 mM KNO_3_ in distilled water.

Presoaking *S. viridis* seeds with GA_3_ at 29°C to 31°C for 24 hours enhanced their germination rates compared to the untreated control seeds soaked in distilled water (Table S1). Although a high concentration of GA_3_ in itself is efficient, a combination of GA_3_ with KNO_3_ more effectively reduced dormancy (Table S1). This combined treatment (2.89 mM GA_3_, 30 mM KNO_3_) was effective even with seeds that were 6 dph (72±3.6% germination compared to 1.66±1.3% in control treated seeds). Treatment of seeds with 2.16 or 2.89 mM GA_3_ supplemented with 30 mM KNO_3_ resulted in 63.6±2.6% and 92.7±3.9% germination, respectively, compared to 6±3.5% in control treated seeds that were presoaked in water (Figure 2A). Lowering the concentration of GA_3_ or increasing the number of seeds treated per mL of treatment solution used reduced the effectiveness of the treatment (Figure 2B & C). Based on our results, it appeared that a ratio close to 10 seeds per 200 µl of GA_3_, KNO_3_ solution worked best. Our results are in general agreement with studies that used GA_3_ to break seed dormancy in other plant species [17], [25]–[28].

### Effect of liquid smoke on dormancy in *S. viridis* seeds

**Figure 3:**
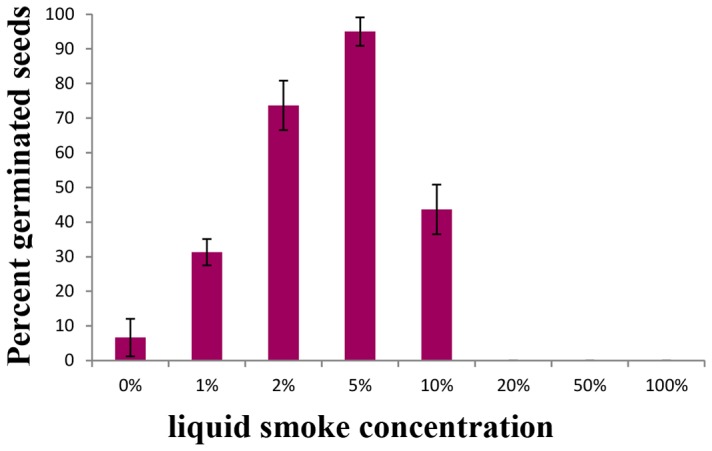
Liquid smoke promotes *S. viridis* seed germination. (**A**) Dose-dependent effect of liquid smoke on stimulating *S. viridis* seed germination (n = 25 seeds per replicate, seed age 30 dph). Error bars represent the standard error.

Smoke and char produced from burning vegetation generates a complex mixture of compounds that act as potent stimulants of seed germination in a number of plant species [29]–[32]. We tested three different commercially available liquid smoke brands to determine if any of them would promote germination in *S. viridis* and found Wright's Liquid Smoke (Hickory) was most effective (Table S3). Seeds of different ages (6 days to 1 month post-harvest) were soaked in an aqueous solution of liquid smoke diluted in distilled water and incubated at 29°C to 31°C for 24 hours. Among the different concentrations of liquid smoke tested, 2% and 5% were the most effective (Figure 3). Use of 5% liquid smoke promotes 90 percent or more germination rates irrespective of seed age between 12 days and one month post-harvest compared to 3.3±2.7% to 16±6.8%, respectively, in control seeds that were soaked in water (Figure 2B). Consistent with the effect of liquid smoke, treating seeds with karrikinolide, one active component of liquid smoke, also promoted germination (Table S1; [33]).

### Effect of GA_3_ and liquid smoke on different accessions of *S. viridis* seeds

**Figure 4:**
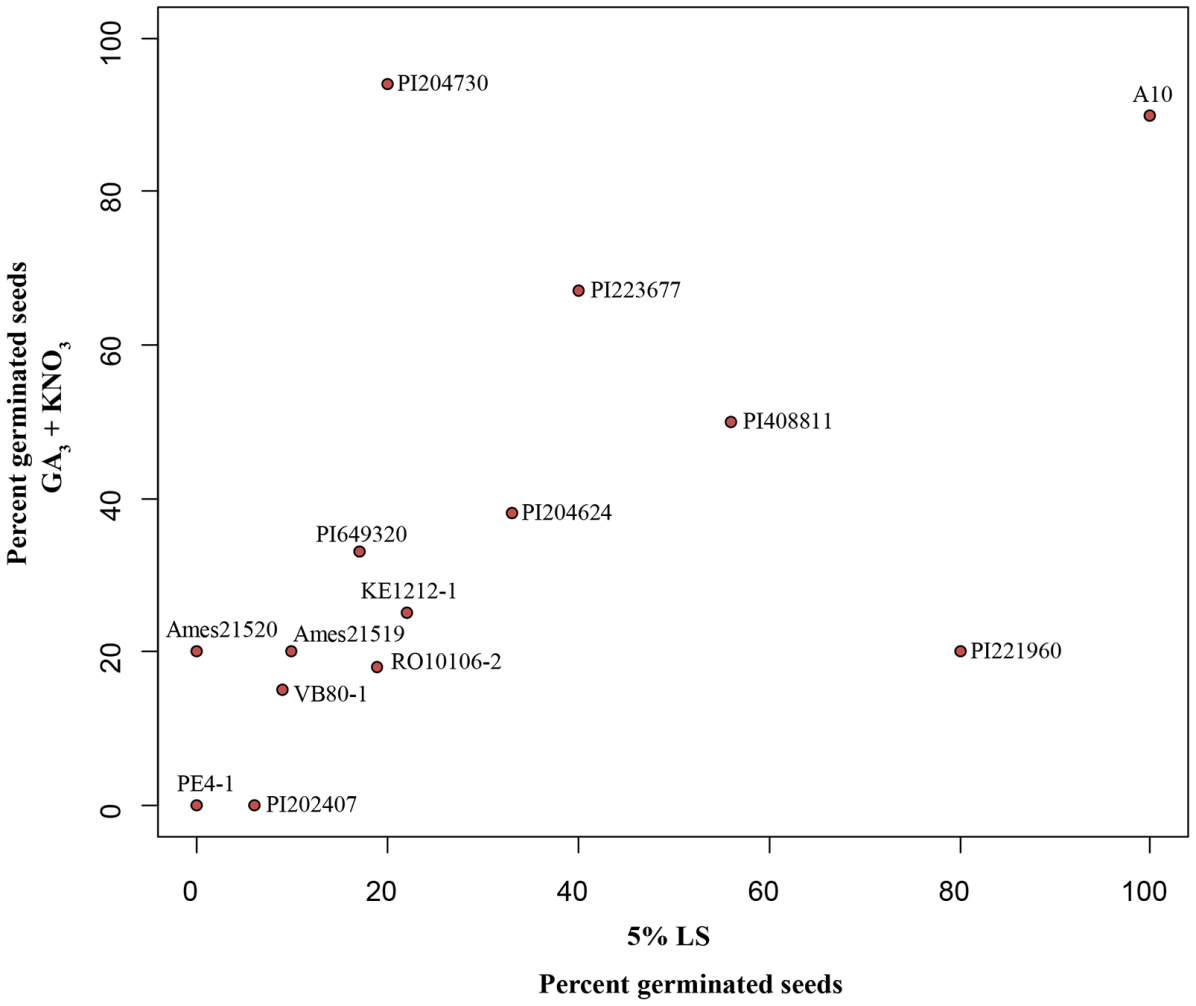
Effect of GA_3_ and liquid smoke on breaking seed dormancy in different *S. viridis* accessions. Effect of GA_3_ and liquid smoke on promoting seed germination in 13 different *S. viridis* accessions (n = 20 to 30 seeds). Graph is plotted using the difference in percent germination observed between the control seeds (incubated in water at 29°C for 24 hours) and GA_3_ + KNO_3_ or liquid smoke treated seeds (seed age 30 to 65 dph).

We next tested the effect of GA_3_ and liquid smoke treatments on a set of geographically diverse *S. viridis* accessions. We performed an initial survey of 17 accessions (Figure S2). Repetition of this survey using an independent batch of seeds revealed consistent effects of GA_3_, KNO_3_ and 5% liquid smoke solution treatments (30°C for 24 hours) for 13 accessions (Figure 4). Interestingly, we observed differences among the accessions in their response to GA_3_ and liquid smoke with several responding to only one treatment but not both (e.g. PI204730, PI221960). These data suggest that there may be extensive genetic diversity in the control of seed dormancy in *S. viridis* accessions, which may be influenced by the local ecological niche the genotype is adapted to.

## Discussion

### An easy, effective and inexpensive method for breaking dormancy in *S. viridis* seeds

*S. viridis* represents a very promising model genetic system to explore diverse aspects of monocot plant growth and development. Although, many of its growth characteristics are ideal for a model genetic system, seed dormancy is a major hindrance in performing experiments rapidly. Seed dormancy is considered an important adaptation of *S. viridis* allowing it to form and maintain a heterogeneous soil seed pool [12]. It has been proposed that factors including varying soil oxygen, water and temperature signals are involved in regulating seed dormancy in *S. viridis* (reviewed by [13]). In this study, our results show that by presoaking the seeds with germination stimulants such as liquid smoke and GA_3_ one could break this long dormancy period in *S. viridis*. We found that GA_3_, KNO_3_ and liquid smoke treatments are effective in breaking dormancy irrespective of seed age and promote germination in seeds as early as 6 dph. A detailed phenotypic characterization of plants from seeds that are germinated using GA_3_, KNO_3_ and liquid smoke appeared normal in growth characteristics. Both of these germination stimulants, especially liquid smoke, are inexpensive and commercially available. Moreover, the seed treatments are easy to perform and can easily be applied to large-scale experiments.

### ABA∶GA_3_ hormone balance and seed germination

Among the multitude of factors that control seed dormancy and germination, the ratio between the phytohormones abscisic acid (ABA) and GA_3_ content in seeds is of major significance. ABA is an inhibitor of seed germination and its accumulation during embryo maturation is known to trigger seed dormancy in many plant species (reviewed by [34]). Consistently, mutant seeds that are ABA-deficient show a decline in dormancy [35], [36]. On the other hand, antagonistic to ABA, GA_3_ is a potent stimulant of seed germination in a number of plant species [37]–[39]. GA_3_ is known to activate various processes associated with seed germination including loosening of the seed coat, expansion and development of the embryo and mobilization of stored nutrient reserves [34]. Thus, the onset of germination versus dormancy of a seed is dependent on the ratio of ABA and GA_3_ present in the seed. Consistent with this, our findings indicated that ABA and GA_3_ had similar effects on dormancy in *S. viridis*. *S. viridis* seeds treated with the ABA biosynthesis inhibitor fluridone or GA_3_ both showed enhanced germination. Thus, taken together, our data suggest that ABA biosynthesis or enhanced sensitivity to ABA might be responsible for its extensive dormancy. Whether treatment with GA_3_ inhibits ABA biosynthesis or ABA signaling to promote germination in *S. viridis* will be an interesting question for future research.

## Supporting Information

Figure S1 **Growth characteristics of plants from seeds treated with GA_3_ and liquid smoke.** (**A, B**) Comparison of plant height (12 DAS) (**A**) and tillering rate (24 DAS) (**B**) in *S. viridis* plants from seeds that are untreated, treated with GA_3_ + KNO_3_ or liquid smoke (n = 20 plants per replicate). (**C**) Rate of leaf initiation in plants from seeds that are untreated, treated with GA_3_ + KNO_3_ or liquid smoke. Only the leaves on the main plant are included for measurement. Plants were grown in D60 pots with a cell diameter of 2.5 inches and a depth of 14 inches (http://www.stuewe.com). All visible tillers present on the main plant are considered for counting. Error bars represent the standard error.
(TIF)

Figure S2 **Initial survey of effect of GA_3_ and liquid smoke on breaking seed dormancy in different** ***S. viridis*** **accessions.** Effect of GA_3_ and liquid smoke on promoting seed germination in 17 different *S. viridis* accessions (n = 10 seeds). Graph is plotted using the difference in percent germination observed between the control seeds (incubated in water at 29°C for 24 hours) and GA_3_ + KNO_3_ or liquid smoke treated seeds (seed age 30 to 40 dph).
(TIF)

Table S1 **Comparison of the effect of various treatments on the rate of** ***S. viridis*** **seed germination.** A comparative list of chemicals and their effect on the rate of *S. viridis* seed germination (seed age 30 dph, n = 10 to 20 seeds). * Seeds without lemma and palea are referred to as naked.
(DOC)

Table S2 **Effect of liquid smoke and GA_3_ + KNO_3_ treatments on** ***S. viridis*** **seed germination determined in tissue culture.** Comparison of the effects of liquid smoke and GA_3_ + KNO_3_ treatments and different seed sterilization protocols on enhancing germination in *S. viridis* seeds on tissue culture plates (seed age 30 dph, n = 20 to 25 seeds per replicate).
(DOC)

Table S3 **Different brands of liquid-smoke and germination rates in** ***S. viridis*** **seeds.** Effect of different commercial liquid-smoke brands and concentrations on germination in *S. viridis* seeds (seed age 30 dph, n = 15 to 20 seeds).
(DOC)
