# Supplementary figures and images for: Methods to Promote Germination of Dormant Setaria viridis Seeds
Source: PLoS One. 2014 Apr 18;9(4):e95109. doi: 10.1371/journal.pone.0095109 (PMC3991590; doi:10.1371/journal.pone.0095109)

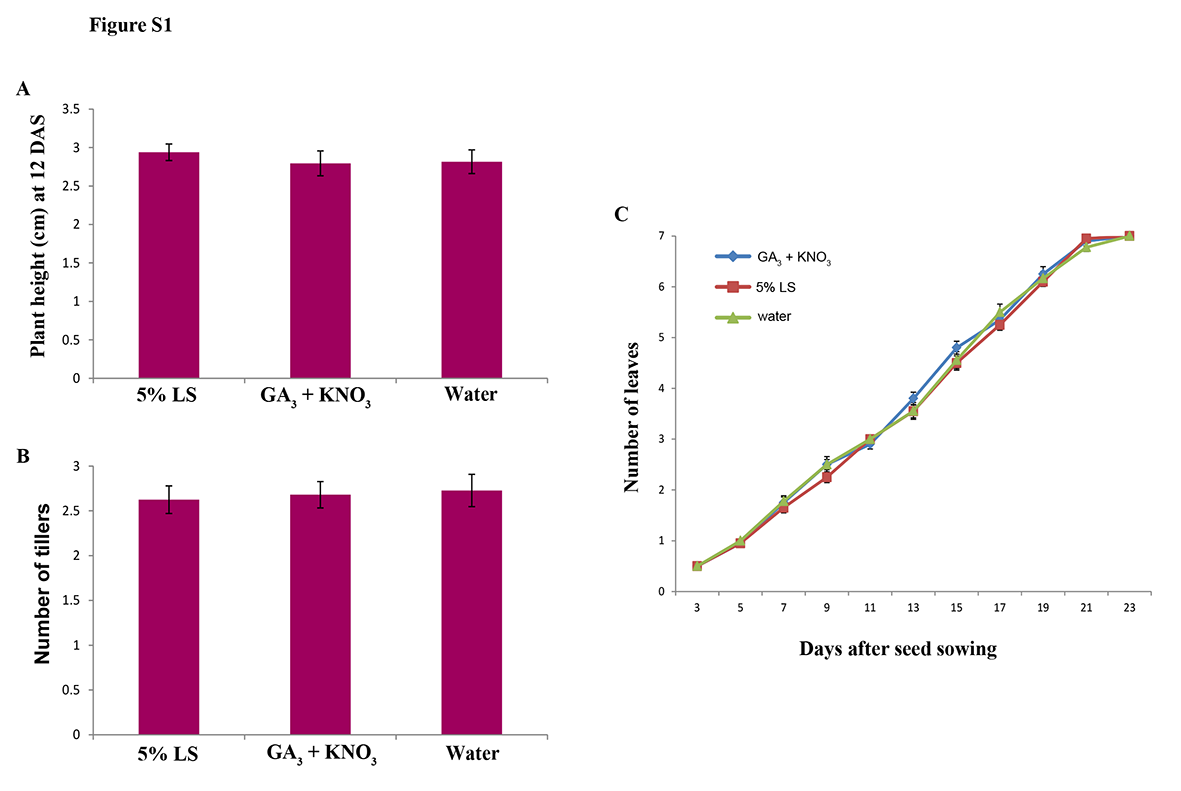

Supplement: Figure S1 — Growth characteristics of plants from seeds treated with GA3 and liquid smoke. (A, B) Comparison of plant height (12 DAS) (A) and tillering rate (24 DAS) (B) in S. viridis plants from seeds that are untreated, treated with GA3 + KNO3 or liquid smoke (n = 20 plants per replicate). (C) Rate of leaf initiation in plants from seeds that are untreated, treated with GA3 + KNO3 or liquid smoke. Only the leaves on the main plant are included for measurement. Plants were grown in D60 pots with a cell diameter of 2.5 inches and a depth of 14 inches (http://www.stuewe.com). All visible tillers present on the main plant are considered for counting. Error bars represent the standard error. (TIF) [file pone.0095109.s001.tif]

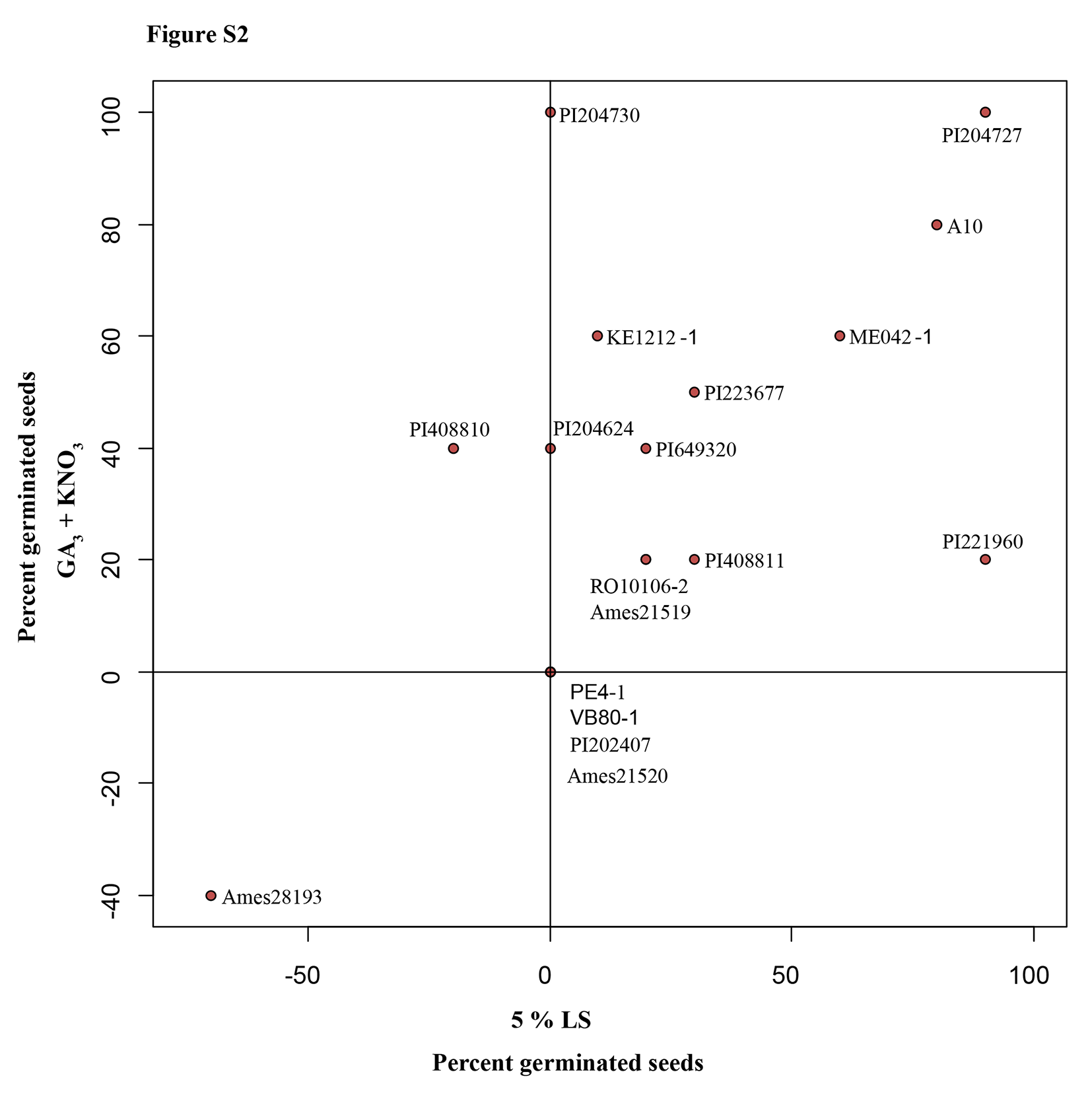

Supplement: Figure S2 — Initial survey of effect of GA3 and liquid smoke on breaking seed dormancy in different S. viridis accessions. Effect of GA3 and liquid smoke on promoting seed germination in 17 different S. viridis accessions (n = 10 seeds). Graph is plotted using the difference in percent germination observed between the control seeds (incubated in water at 29°C for 24 hours) and GA3 + KNO3 or liquid smoke treated seeds (seed age 30 to 40 dph). (TIF) [file pone.0095109.s002.tif]
