# Supplementary material for: Methods to Promote Germination of Dormant Setaria viridis Seeds
Source: PLoS One. 2014 Apr 18;9(4):e95109. doi: 10.1371/journal.pone.0095109 (PMC3991590; doi:10.1371/journal.pone.0095109)
Supplement: Table S1 — Comparison of the effect of various treatments on the rate of S. viridis seed germination. A comparative list of chemicals and their effect on the rate of S. viridis seed germination (seed age 30 dph, n = 10 to 20 seeds). * Seeds without lemma and palea are referred to as naked. (DOC) [file pone.0095109.s003.doc]

**Table S1**

| Treatments and conditions | Percent germinated seeds |
| --- | --- |
| 10 mM KNO_3_ at 29⁰C for 24 hours | 0% |
| 30 mM KNO_3_ at 29⁰C for 24 hours | 20% |
| 50 mM KNO_3_ at 29⁰C for 24 hours | 20% |
| 10 mM KNO_3_ at 29⁰C for 120 hours | 0% |
| 50 mM KNO_3_ at 4⁰C for 120 hours | 0% |
| 2 mM CaSO_4_ at 29⁰C for 24 hours | 20% |
| 2 mM CaSO_4_ with 30 mM KNO_3_ at 29⁰C for 24 hours | 20% |
| 6 mM CaSO_4_ at 29⁰C for 24 hours | 0% |
| 6 mM CaSO_4_ with 30 mM KNO_3_ at 29⁰C for 24 hours | 20% |
| 2 mM CaSO_4_ at 29⁰C for 120 hours | 0% |
| 2 mM CaSO_4_ with 30 mM KNO_3_ at 29⁰C for 120 hours | 20% |
| 6 mM CaSO_4_ at 29⁰C for 120 hours | 0% |
| 6 mM CaSO_4_ with 30 mM KNO_3_ at 29⁰C for 120 hours | 20% |
| Thermal shock at 90.5⁰C for 90 seconds; incubated in H_2_O for 24 hours | 0% |
| Thermal shock at 95⁰C for 5 minutes; incubated in H_2_O for 24 hours | 0% |
| Thermal shock at 90.5⁰C for 90 seconds; incubated in H_2_O for 120 hours | 0% |
| Thermal shock at 95⁰C for 5 minutes; incubated in H_2_O for 120 hours | 0% |
| Shaking in water at 30⁰C for 7 days | 0% |
| Shaking in water at 30⁰C for 14 days | 0% |
| Cold treatment with soil at 4⁰C for 8 days | 6% |
| Cold treatment with soil at 4⁰C for 14 days | 55% |
| 0.01 µM Karrikinolide at 4⁰C with light for 24 hours | 25% |
| 0.01 µM Karrikinolide at 4⁰C without light for 24 hours | 25% |
| 0.01 µM Karrikinolide at 29⁰C with light for 24 hours | 0% |
| 0.01 µM Karrikinolide at 29⁰C without light for 24 hours | 25% |
| 0.01 µM Karrikinolide at 4⁰C with light for 144 hours | 0% |
| 0.01 µM Karrikinolide at 4⁰C without light for 144 hours | 0% |
| 0.01 µM Karrikinolide at 29⁰C with light for 144 hours | 0% |
| 0.01 µM Karrikinolide at 29⁰C with light for 144 hours | 0% |
| 0.5 µM Karrikinolide at 4⁰C with light for 24 hours | 0% |
| 0.5 µM Karrikinolide at 4⁰C without light for 24 hours | 0% |
| 0.5 µM Karrikinolide at 29⁰C with light for 24 hours | 50% |
| 0.5 µM Karrikinolide at 29⁰C without light for 24 hours | 25% |
| 0.5 µM Karrikinolide at 4⁰C with light for 144 hours | 25% |
| 0.5 µM Karrikinolide at 4⁰C without light for 144 hours | 0% |
| 0.5 µM Karrikinolide at 29⁰C with light for 144 hours | 25% |
| 0.5 µM Karrikinolide at 29⁰C without light for 144 hours | 25% |
| 1 µM Karrikinolide at 4⁰C with light for 24 hours | 0% |
| 1 µM Karrikinolide at 4⁰C without light for 24 hours | 25% |
| 1 µM Karrikinolide at 29⁰C with light for 24 hours | 80% |
| 1 µM Karrikinolide at 29⁰C without light for 24 hours | 25% |
| 2 µM Karrikinolide at 29⁰C with light for 24 hours | 60% |
| 1 µM Karrikinolide at 4⁰C with light for 144 hours | 0% |
| 1 µM Karrikinolide at 4⁰C without light for 144 hours | 0% |
| 1 µM Karrikinolide at 29⁰C with light for 144 hours | 50% |
| 1 µM Karrikinolide at 29⁰C without light for 144 hours | 25% |
| 5% Colgin liquid smoke (Hickory) at 29⁰C for 2 hours | 10% |
| 5% Colgin liquid smoke (Hickory) at 29⁰C for 24 hours | 80% |
| Naked seeds 289 µM GA_3_ at 29⁰C for 24 hours | 10% |
| Naked seeds 289 µM GA_3_ with 30 mM KNO_3_ at 29⁰C for 24 hours | 20% |
| Naked seeds 289 µM GA_3_ with 30 mM KNO_3_ at 29⁰C for 120 hours | 0% |
| 289 µM GA_3_ with 30 mM KNO_3_ at 29⁰C for 120 hours | 30% |
| 289 µM GA_3_ at 29⁰C for 120 hours | 0% |
| Naked seeds 289 µM GA_3_ at 29⁰C for 120 hours | 0% |
| 289 µM GA_3_ at 29⁰C for 24 hours | 10% |
| 289 µM GA_3_ 30 mM KNO_3_ at 29⁰C for 24 hours | 20% |
| 1.44 mM GA_3_ at 29⁰C for 24 hours | 30% |
| Naked seeds 1.44 mM GA_3_ at 29⁰C for 24 hours | 30% |
| 1.44 mM GA_3_ with 30mM KNO_3_ at 29⁰C for 24 hours | 40% |
| Naked seeds 1.44 mM GA_3_ with 30 mM KNO_3_ at 29⁰C for 24 hours | 0% |
| Naked seeds 1.44 mM GA_3_ at 29⁰C for 120 hours | 0% |
| Naked seeds 1.44 mM GA_3_ with 30 mM KNO_3_ at 29⁰C for 120 hours | 0% |
| 1.44 mM GA_3_ at 29⁰C for 120 hours | 40% |
| Naked seeds 144 µM GA_3_ at 29⁰C for 120 hours | 0% |
| 2.89 mM GA_3_ at 29⁰C for 24 hours | 50% |
| 2.89 mM GA_3_ at 29⁰C for 120 hours | 50% |
| 2.89 mM GA_3_ with 30 mM KNO_3_ at 29⁰C for 24 hours | 100% |
| 2.89 mM GA_3_ with 30 mM KNO_3_ at 4⁰C for 24 hours | 20% |
| Naked seeds 2.89 mM GA_3_ with 30 mM KNO_3_ at 29⁰C for 24 hours | 30% |
| Naked seeds 2.89 mM GA_3_ with 30 mM KNO_3_ at 4⁰C for 24 hours | 60% |
| 2.89 mM GA_3_ with 30 mM KNO_3_ at 29⁰C for 120 hours | 50% |
| 2.89 mM GA_3_ with 30 mM KNO_3_ at 4⁰C for 120 hours | 20% |
| Naked seeds 2.89 mM GA_3_ with 30 mM KNO_3_ at 4⁰C for 120 hours | 0% |
| Naked seeds 2.89 mM GA_3_ with 30 mM KNO_3_ at 29⁰C for 120 hours | 20% |
| 100 µM Fluridone at 4⁰C for 24 hours | 40% |
| 100 µM Fluridone at 29⁰C for 24 hours | 20% |
| 100 µM Fluridone with 30 mM KNO_3_ at 29⁰C for 24 hours | 60% |
| 100 µM Fluridone at 29⁰C for 120 hours | 70% |
| 100 µM Fluridone at 4⁰C for 120 hours | 20% |
| 100 µM Fluridone with 30 mM KNO_3_ at 29⁰C for 120 hours | 80% |
| Naked seeds 100 µM Fluridone 30 mM KNO_3_ at 29⁰C for 24 hours | 10% |
| Naked seeds 100 µM Fluridone 30 mM KNO_3_ at 29⁰C for 120 hours | 0% |
| 30 µM Fluridone at 29⁰C for 24 hours | 0% |
| Naked seeds 30 µM Fluridone at 29⁰C for 24 hours | 0% |
| 10 µM Fluridone with 30 mM KNO_3_ at 29⁰C for 120 hours | 30% |
| Water at 4⁰C for 24 hours | 0% |
| Water at 4⁰C for 7 days | 10% |
| At -80⁰C for 3 days | 10% |
| Naked seeds water at 29⁰C for 24 hours | 0% |
| Naked seeds in water at 4⁰C for 24 hours | 0% |
| Naked seeds in water at 29⁰C for 7 days | 0% |
