# Supplementary material for: Methods to Promote Germination of Dormant Setaria viridis Seeds
Source: PLoS One. 2014 Apr 18;9(4):e95109. doi: 10.1371/journal.pone.0095109 (PMC3991590; doi:10.1371/journal.pone.0095109)
Supplement: Table S2 — Effect of liquid smoke and GA3 + KNO3 treatments on S. viridis seed germination determined in tissue culture. Comparison of the effects of liquid smoke and GA3 + KNO3 treatments and different seed sterilization protocols on enhancing germination in S. viridis seeds on tissue culture plates (seed age 30 dph, n = 20 to 25 seeds per replicate). (DOC) [file pone.0095109.s004.doc]

**Table S2**

| ­­­­Treatment | Sterilization | Percent germinated seeds |
| --- | --- | --- |
| Wright's Hickory 0.1% at 29°C for 24 hours | Ethanol 95% for 10 minutes | 0% |
| Wright's Hickory 0.5% at 29°C for 24 hours | Ethanol 95% for 10 minutes | 10% |
| Wright's Hickory 1% at 29°C for 24 hours | Ethanol 95% for 10 minutes | 20% |
| Wright's Hickory 0.1% at 29°C for 24 hours | Bleach 20% for 20 minutes | 60% |
| Wright's Hickory 0.5% at 29°C for 24 hours | Bleach 20% for 20 minutes | 50% |
| Wright's Hickory 1% at 29°C for 24 hours | Bleach 20% for 20 minutes | 80% |
| Wright's Hickory 2% at 29°C for 24 hours | Bleach 20% for 20 minutes | 20% |
| Wright's Hickory 2% at 29°C for 24 hours (naked seeds) | Bleach 20% for 20 minutes | 0% |
| Wright's Hickory 5% at 29°C for 24 hours | Bleach 20% for 20 minutes | 0% |
| Wright's Hickory 5% at 29°C for 30 minutes | Bleach 20% for 20 minutes | 33% |
| Wright's Hickory 10% at 29°C for 30 minutes | Bleach 20% for 20 minutes | 0% |
| 2.89 mM GA_3_ with 30 mM KNO_3_ at 29°C for 24 hours | Bleach 20% for 20 minutes | 90% |
| 2.16 mM GA_3_ with 30 mM KNO_3_ at 29°C for 24 hours | Bleach 20% for 20 minutes | 80% |
| 1.44 mM GA_3_ with 30 mM KNO_3_ at 29°C for 24 hours | Bleach 20% for 20 minutes | 78% |
| Water at 29°C for 24 hours | Bleach 20% for 20 minutes | 8.6% |
