# Supplementary material for: Methods to Promote Germination of Dormant Setaria viridis Seeds
Source: PLoS One. 2014 Apr 18;9(4):e95109. doi: 10.1371/journal.pone.0095109 (PMC3991590; doi:10.1371/journal.pone.0095109)
Supplement: Table S3 — Different brands of liquid-smoke and germination rates in S. viridis seeds. Effect of different commercial liquid-smoke brands and concentrations on germination in S. viridis seeds (seed age 30 dph, n = 15 to 20 seeds). (DOC) [file pone.0095109.s005.doc]

**Table S3**

| Treatment and conditions | Percent germinated seeds |
| --- | --- |
| 0.5% Wright’s Hickory at 29⁰C for 30 minutes | 10% |
| 0.5% Wright’s Hickory at 29⁰C for 2 hours | 30% |
| 0.5% Wright’s Hickory at 29⁰C for 24 hours | 20% |
| 1% Wright’s Hickory at 29⁰C for 30 minutes | 20% |
| 1% Wright’s Hickory at 29⁰C for 2 hours | 10% |
| 1% Wright’s Hickory at 29⁰C for 24 hours | 0% |
| 5% Wright’s Hickory at 29⁰C for 30 minutes | 10% |
| 5% Wright’s Hickory at 29⁰C for 2 hours | 10% |
| 5% Wright’s Hickory at 29⁰C for 24 hours | 100% |
| 50% Wright’s Hickory at 29⁰C for 30 minutes | 75% |
| 50% Wright’s Hickory at 29⁰C for 24 hours | 0% |
| 100% Wright’s Hickory at 29⁰C for 30 minutes | 50% |
| 100% Wright’s Hickory at 29⁰C for 2 hours | 0% |
| 100% Wright’s Hickory at 29⁰C for 24 hours | 0% |
| 0.5% Colgin Hickory at 29⁰C for 30 minutes | 20% |
| 0.5% Colgin Hickory at 29⁰C for 2 hours | 20% |
| 0.5% Colgin Hickory at 29⁰C for 24 hours | 0% |
| 5% Colgin Hickory at 29⁰C for 30 minutes | 20% |
| 5% Colgin Hickory at 29⁰C for 2 hours | 0% |
| 5% Colgin Hickory at 29⁰C for 24hours | 77% |
| 100% Colgin Hickory at 29⁰C for 30 minutes | 44% |
| 100% Colgin Hickory at 29⁰C for 2 hours | 20% |
| 100% Colgin Hickory at 29⁰C for 24 hours | 0% |
| 100% Colgin Hickory at 29⁰C for 30 minutes | 40% |
| 100% Colgin Hickory at 29⁰C for 2 hours | 20% |
| 100% Colgin Hickory at 29⁰C for 24 hours | 0% |
| 0.5% Colgin Mesquite at 29⁰C for 30 minutes | 10% |
| 0.5% Colgin Mesquite at 29⁰C for 2 hours | 0% |
| 0.5% Colgin Mesquite at 29⁰C for 24 hours | 0% |
| 5% Colgin Mesquite at 29⁰C for 30 minutes | 5% |
| 5% Colgin Mesquite at 29⁰C for 2 hours | 0% |
| 5% Colgin Mesquite at 29⁰C for 24 hours | 34% |
| 100% Colgin Mesquite at 29⁰C for 30 minutes | 20% |
| 100% Colgin Mesquite at 29⁰C for 2 hours | 10% |
| 100% Colgin Mesquite at 29⁰C for 24 hours | 0% |
| 5% Colgin Hickory with 30 mM KNO_3_ at 29⁰C for 24 hours | 60% |
| 100% Colgin Hickory with 30 mM KNO_3_ at 29⁰C for 2 hours | 0% |
| 100% Colgin Hickory with 30 mM KNO_3_ at 29⁰C for 24 hours | 0% |
| 5% Colgin Mesquite with 30 mM KNO_3_ at 29⁰C for 2 hours | 20% |
| 5% Colgin Mesquite with 30 mM KNO_3_ at 29⁰C for 24 hours | 40% |
| 100% Colgin Mesquite with 30 mM KNO_3_ at 29⁰C for 2 hours | 20% |
| 100% Colgin Mesquite with 30 mM KNO_3_ at 29⁰C for 24 hours | 0% |
| Water at 29⁰C for 24 hours | 20% |
| Water at 4⁰C for 24 hours | 0% |
| 6 dph seeds in 5% Wright’s Hickory at 29⁰C for 24 hours | 68% |
| 6 dph seeds in water at 29⁰C for 24 hours | 1.6% |
